# How intrasexual competitiveness shapes attitudes towards cosmetic surgery recipients

**DOI:** 10.1017/ehs.2023.26

**Published:** 2023-11-09

**Authors:** Sarah Bonell, Christoph Klebl, Khandis Blake, Scott Griffiths

**Affiliations:** 1 Department of Psychology, Royal Melbourne Institute of Technology, Melbourne, Australia; 2 School of Psychology, The University of Queensland, Brisbane, Australia; 3 Melbourne Schoolof Psychological Sciences, The University of Melbourne, Melbourne, Australia

**Keywords:** cosmetic surgery, plastic surgery, intrasexual competitiveness, feminism, body dysmorphic disorder

## Abstract

Cosmetic surgery is extremely popular. Despite this, negative attitudes towards cosmetic surgery recipients prevail. Across two pre-registered studies, we examined whether intrasexual competitiveness explains these negative attitudes. Participants in Study 1 were 343 (mean age = 24.74) single heterosexual American women and participants in Study 2 were 445 (mean age = 19.03) single heterosexual Australian women. Participants in both studies were primed for either low or high intrasexual competitiveness. Contrary to our predictions, we found that priming condition did not influence participants’ derogation and social exclusion of cosmetic surgery recipients. We did, however, find evidence for a ‘relative attractiveness’ halo effect: participants engaged in less derogation and social exclusion when they assumed cosmetic surgery recipients were more attractive than themselves. This suggests that 'pretty privilege' extends not only to women who meet conventional beauty standards, but also to those who are perceived as *relatively closer to meeting these standards* than the individual with whom they are engaging. Overall, we concluded that intrasexual competitiveness does not encourage the stigmatisation of cosmetic surgery recipients and examined alternative explanations for this phenomenon.

## Graphical abstract

**Figure:**
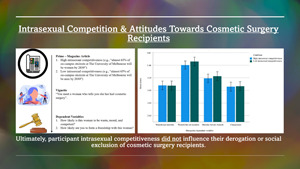

**Social media summary**: The stigmatisation of cosmetic surgery is pervasive, but findings suggest intrasexual competitiveness is not to blame.

## Introduction

Cosmetic surgery is a popular form of appearance modification, with over 18 million cosmetic surgery procedures performed in the US each year. This multibillion-dollar industry is subsidised largely by women, who account for approximately 92% of all recipients (American Society of Plastic Surgeons, 2018, 2019). Typically, women who choose to undergo cosmetic surgery report feeling pleased with their surgical results and demonstrate greater appearance satisfaction up to 5 years post-surgery (Honigman et al., 2004; Sharp et al., 2017; von Soest et al., 2011). As such, cosmetic surgery serves as a means through which women can become more conventionally attractive. Physical attractiveness heavily influences our perceptions of people; those who are attractive are consistently perceived as more likeable, capable, intelligent (Dion et al., 1972; Eagly et al., 1991; Wheeler & Kim, 1997), moral (Klebl et al., 2022) and human (Bonell et al., 2021a, 2021b). These findings suggest that greater attractiveness via cosmetic procedures ought to, if anything, improve perceptions of cosmetic surgery recipients.

### Perceptions of cosmetic surgery recipients

Despite the link between attractiveness and positive perceptions, perceptions of women are actually worsened when they choose to undergo cosmetic surgery. For example, Saxena (2013) demonstrated that cosmetic surgery recipients often feel they are negatively stereotyped as fake, vain, insecure, unintelligent, and unstable. Similarly, both Delinsky (2005) and Tam et al. (2012) found that most people demonstrate disapproval towards cosmetic surgery and its recipients. Finally, Bonell et al. (2021b) investigated whether perceptions of cosmetic surgery recipients systematically differed from perceptions of non-recipients. Results demonstrated that women planning to undergo cosmetic surgery were rated less favourably than those planning to engage in control activities across warmth, competence, morality and humanness. Overall, these results suggest that cosmetic surgery recipients are derogated (i.e. belittled, diminished) and socially excluded (i.e. avoided, ‘othered’). What remains unclear, however, are the motivations that elicit negative attitudes towards cosmetic surgery recipients. The purpose of this paper will be to examine one potential mechanism through which negative cosmetic surgery attitudes are born: intrasexual competitiveness.

### Intrasexual competitiveness and cosmetic surgery

Until recently, the privileges afforded to attractive women were only made available to those lucky enough to be born beautiful. With the rise of cosmetic surgery, however, beauty is slowly becoming more attainable; for those who can afford it, beauty is now a purchasable commodity (Heyes & Jones, 2016). Studies in which participants have rated cosmetic surgery recipients on their attractiveness have indicated that mean attractiveness scores are indeed higher for women after receiving cosmetic surgery (Nellis et al., 2017, 2018; Reilly et al., 2015). Given the importance men place on physical beauty when selecting partners, cosmetic surgery might therefore afford heterosexual women improved chances of forming long-term partnerships with men (Eastwick & Finkel, 2008; Grammer et al., 2003). However, as each and every woman has access to only a finite number of potential male partners, by the very nature of using cosmetic surgery to become more attractive, cosmetic surgery recipients diminish non-recipients’ chances of acquiring partners. In other words, beauty (and its associated privileges) is inherently something that can be ‘stolen’ – as one woman undergoes cosmetic surgery to become more beautiful, another becomes less beautiful by comparison (Blum, 2003). We therefore propose that, among women, cosmetic surgery recipients are perceived negatively because of rivalry between women over access to mates. Below we explain the various factors that may shape the relationship between intrasexual competitiveness and negative perceptions of cosmetic surgery recipients.

### Relative attractiveness of the recipient

Given that attractiveness is predictive of mating success for heterosexual women (Buss, 1989; Eastwick & Finkel, 2008; Grammer et al., 2003), we contend that the more attractive a cosmetic surgery recipient is, the greater threat she poses to non-recipients. However, this threat effect is also relative to the attractiveness of the non-recipient in question. In other words, if a woman undergoes cosmetic surgery and, as a result, ends up looking ‘average’ (based on coventional heteronormative beauty standards), she may not pose a threat to a non-recipient who considers herself attractive; however, if this same recipient becomes extremely attractive after undergoing surgery, she will pose a threat to the attractive non-recipient. Therefore, if intrasexual competitiveness does indeed influence attitudes towards cosmetic surgery recipients, we might expect the relative attractiveness of recipients (that is, how attractive they are compared with participants’ own attractiveness) to moderate this effect.

### Sex-is-power belief

While studies show that men value physical attractiveness highly when assessing potential partners, this does not necessarily mean that all women *believe* that attractiveness plays an integral role in their ability to find a male partner (Eastwick & Finkel, 2008; Grammer et al., 2003). The sex-is-power belief denotes the belief that women are able to use their sexual appeal to gain power over men (Erchull & Liss, 2013). We contend that women who believe that sex-is-power might find the idea of a surgically enhanced (and arguably more attractive) woman to be more threatening than women who believe that other factors (e.g. their intelligence or humour) play a greater role in attracting a mate. Our reasoning is that women who believe sex-is-power may inherently place more value on beauty as a means for mating competition. We therefore propose that participants' sex-is-power beliefs will moderate the degree to which intrasexual competitiveness influences cosmetic surgery perceptions.

## Study aims and hypotheses

We examine the relationship between intrasexual competitiveness, perceptions of cosmetic surgery recipients, sex-is-power belief, and relative attractiveness. We hypothesise that participants in the high intrasexual competitiveness condition will derogate and socially exclude cosmetic surgery recipients more than those in the low intrasexual competitiveness condition. We also propose two secondary, exploratory hypotheses: the relationships between prime condition and derogation, and prime condition and social exclusion, will be moderated by (i) the relative attractiveness of the recipient in question and (ii) the participant's sex-is-power belief. Namely, the positive relationships between intrasexual competitiveness and derogation and social exclusion will be *intensified* for participants who (i) consider the cosmetic surgery recipient more relatively attractive and (ii) hold stronger sex-is-power beliefs.

### Changes from pre-registration

As per our pre-registration (https://tinyurl.com/2pymy5z6), we initially intended to conduct only one study for this paper (see Study 1). However, we decided to conduct an additional study (see Study 2) to address methodological flaws discovered in Study 1.

## Study 1

### Method

#### Participants

Ethics approval was obtained from The Psychological Sciences Human Ethics Advisory Group at The University of Melbourne (ID 14058). Participants were 343 (mean age = 24.74, standard deviation, SD = 13.41) single heterosexual American women recruited via Amazon's Mechanical Turk (MTurk). Most participants (82%) identified as White (*N* = 280; including multiracial participants who identified in part as White). Compensation for each participant completing our 20 minute survey was USD 2.70. We excluded 39 participants for failing the attention check (see Measures).

#### Measures

##### Prime condition

Participants were primed for intrasexual competitiveness (levels: low, high) using fictitious magazine article stimuli sourced from Arthur et al. (2020). These articles influenced women's perceptions of access to mates. They suggested that women in the US had either many (level: low) or few (level: high) options for finding a male romantic partner; that is, the stimulus indicated that the sex ratio in the US was either male-dominated (e.g. “nowadays, there is a flood of eligible bachelors on the market”) or female-dominated (e.g. “experts say that a man drought exists in the US”), respectively.

##### Attention check

As per Arthur et al. (2020), participants were asked to select the best summary of the magazine article they had just read from a list of three potential synopses. Participants who responded incorrectly were excluded from the study. This attention check was not only intended to operate as a means to exclude non-attentive participants, but also as a means through which attentive participants could better engage with and comprehend the magazine article stimulus (see Procedure).

##### Manipulation check: intrasexual competition scale

Participants completed the 12-item Intrasexual Competition Scale (Buunk & Fisher, 2009). They responded to questions on a seven-point Likert scale (1 = *not at all applicable*, 7 = *completely applicable*) designed to assess the degree to which they felt motivated to compete with other women (e.g. “I can't stand it when I meet another woman who is more attractive than I am”). Items were averaged to form a mean score for each participant.

##### Sex-is-power belief

Participants’ sex-is-power beliefs were measured using the five-item Women-Sex is Power Scale (W-SIPS), which demonstrates good internal consistency and validity (Erchull & Liss, 2013). Participants responded to questions on a six-point Likert scale (1 = *disagree strongly*, 7 = *agree strongly*) designed to assess the degree to which they believe that women can use sexual appeal to gain power over men (e.g. “women can use their looks to control men”). Items were averaged to form participant mean scores.

##### Vignette

Participants were presented with a vignette that read: “*you meet a woman who tells you she has had cosmetic surgery*”. A similar vignette has been used successfully in previous literature examining attitudes towards cosmetic surgery recipients (Bonell et al., 2021b).

##### Relative attractiveness of the recipient

Pertaining to the woman described in the vignette, participants were asked: “*compared with your own attractiveness, how attractive do you think this woman is likely to be?*” Responses were given on a five-point Likert scale (1 = *much less attractive than me*, 5 = *much more attractive than me*). This measure is unique in that it is a composite measure of recipient and participant attractiveness – that is, it measures how attractive participants feel the recipient is in relation to themselves (the gap between their own self-perceived attractiveness and the assumed attractiveness of the cosmetic surgery recipient). This is different from a measure of participant attractiveness or recipient attractiveness alone.

##### Negative perceptions

We conceptualised negative perceptions as scores on derogation and social exclusion and examined these as two unique dependent variables.

*Derogation*. As per Bonell et al. (2021b), participants were asked to rate their perceptions of the woman described in the vignette across 32 previously validated personality traits (Goodwin, 2015; Goodwin et al., 2014). These traits were designed to reflect perceptions of warmth, morality and competence. Specifically, eight of these traits reflected perceptions of both warmth and morality (e.g. humble, kind), eight reflected perceived warmth but not morality (e.g. funny, sociable), eight reflected perceived morality but not warmth (e.g. just, principled) and eight reflected perceived competence (e.g. athletic, intelligent). Participants were asked to indicate the likelihood that the woman described in the vignette possessed each of these traits, with ratings given on a five-point Likert scale (1 = *extremely unlikely*, 5 = *extremely likely*). Participants’ average scores were calculated for each subscale independently.

*Social exclusion*. As per Krems et al. (2016), social exclusion was measured using a three-item scale. On a six-point Likert scale, participants indicated (i) the amount of contact they would like to have with the woman described in the vignette (1 = *I wouldn't want any kind of contact*, 6 = *I could see her as a best friend*), (ii) their willingness to consider her a close friend (1 = *very unwilling*, 6 = *very willing*) and (iii) their overall impressions of her (1 = *I strongly dislike her*, 6 = *I very much like her*). Scores were averaged for each participant across the three items.

Please note (as per the response scales outlined above) that derogation and social exclusion are greater/higher when scores on these two measures are *lower*; that is, lower scores indicate greater derogation and social exclusion.

##### Descriptive statistics for measures

See Table 1 for measure descriptive statistics.

**Table 1.** Descriptive statistics for measures included in Study 1

Scale	Subscale	Mean	SD	*α*
**Intrasexual Competition Scale**		3.04	1.45	0.94
**Sex-is-power belief**		4.33	1.09	0.92
**Relative Attractiveness of the Recipient**		3.64	1.00	NA
**Derogation**	Warmth and morality	3.10	0.81	0.93
	Warmth but not morality	3.42	0.67	0.84
	Morality but not warmth	3.20	0.73	0.88
	Competence	3.20	0.73	0.88
**Social exclusion**		4.33	1.09	0.93

#### Procedure

**Figure 1.**
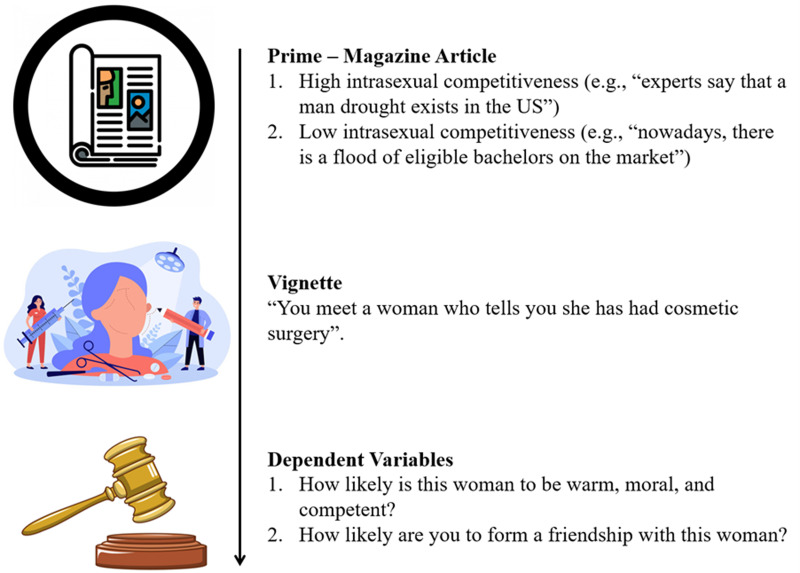
(Abridged) procedure for Study 1.

After providing consent, participants were told they would be presented with a magazine article are were instructed to pay attention to the content of the article. Participants were randomly presented with either the low or high intrasexual competitiveness priming article. To give participants the opportunity to better engage with and comprehend the magazine article stimulus, they were then told that they were about to be asked question(s) pertaining to the article, and were given the option to go back and re-read the article before continuing. Once participants were satisfied that they understood the content of the article, they were presented with the attention check. Next, in order to determine whether our magazine article primes did indeed manipulate participants’ intrasexual competitiveness, participants were presented with the manipulation check (i.e. the Intrasexual Competition Scale). Participants then completed the W-SIPS to assess their sex-is-power belief. At this point, they were presented with the vignette and were asked to indicate the relative attractiveness of the recipient. Participants then completed the derogation measure and the social exclusion measure. Finally, they were presented with a debriefing statement. See Figure 1 for a summary of the major components (i.e., prime and dependent variables) of our procedure.

#### Statistical analyses

To assess whether our intrasexual competitiveness manipulation was successful, a two-tailed independent sample *t*-test was used to detect mean differences of condition (low vs. high intrasexual competition) on the manipulation check (Intrasexual Competition Scale). Because our manipulation was unsuccessful (see Results), we do not report the results of our intended analyses for Study 1. Our intended analyses for Study 1 were identical to those outlined in the ‘Statistical Analyses’ section of Study 2. Manipulation check analyses for Study 1 were carried out using JASP software. Data and code are available on the Open Science Framework (https://tinyurl.com/2pymy5z6).

#### Power analysis

An *a-priori* power analysis was conducted for sample size estimation using G*Power. Assuming a small effect size (*f*^2^ = 0.02), *α* = 0.05, and 80% power (β = 0.80), the analysis indicated that 395 participants were required for our moderation analyses. Accounting for the >5% fail rate for attention checks among MTurk participants (Hauser & Schwarz, 2016; Peer et al., 2017), we concluded that recruiting approximately 415 participants would be ideal.

### Results

#### Manipulation check

We examined whether our manipulation for priming intrasexual competitiveness was successful. Despite the fact that ample existing literature has demonstrated that female intrasexual competition increases in female-biased sex ratio environments (Arnocky et al., 2014; Schacht et al., 2017; Uecker & Regnerus, 2010), our manipulation was unsuccessful. That is, intrasexual competitiveness did not differ between participants in the low (*M* = 2.92, 184, SD = 1.26) and high (*M* = 3.14, SD = 1.56) intrasexual competitiveness prime conditions, *p* = 0.167. Because our manipulation failed, we did not conduct our intended analyses for Study 1.

### Discussion

We contend that there are two potential reasons our manipulation failed in Study 1: the manipulation itself was not sufficient to induce intrasexual competitiveness and/or the Intrasexual Competition Scale was not appropriate for operationalising state-level (i.e. ‘in the moment’) intrasexual competition.

We attempted to prime participants with information regarding sex ratios across the entirety of the US. Perhaps participants were unaffected by our primes because no information pertaining to their immediate social environment was included. That is, we might have induced intrasexual competitiveness more convincingly had the sex ratios provided been relevant to the participants’ hometown, for instance (Arnocky et al., 2014; Schacht et al., 2017; Uecker & Regnerus, 2010). It is also possible that the Intrasexual Competition Scale might not be suitable for measuring state intrasexual competitiveness (vs. trait intrasexual competitiveness) because some items are relevant to measuring expressions of intrasexual competitiveness over time (e.g. “*I always want to beat other women*”) or in context-specific scenarios (e.g. “*When I'm at a party, I enjoy it when men pay more attention to me than other women*”), while others explicitly assess absolute beliefs (e.g. “*I just don't like very ambitious women*”). Because our manipulation check failed, we conducted a second study in which both limitations were addressed.

## Study 2

Study 2 was conducted to address limitations in the manipulation used in Study 1. We made changes to several aspects of methodology (see Participants and Measures), while replicating the analyses conducted in Study 1. We also recruited more participants to ensure we had adequate power to run moderation analyses.

### Method

#### Participants

Participants were 445 (mean age = 19.03, SD = 2.14) female psychology undergraduate students recruited via the Research Experience Program at The University of Melbourne. All participants were single and heterosexual. Including being mixed-race, most participants identified as either Asian/Asian Indian (*N* = 259, 58%) or White (*N* = 172, 39%). No participants from the study were excluded for failing the attention check.

#### Measures

##### Prime condition

Participants were primed for intrasexual competitiveness (levels: low, high) using magazine article stimuli. While in Study 1 we used articles sourced from Arthur et al. (2020), in Study 2 we wrote our own articles. Each article suggested that female students at The University of Melbourne had either many (level: low) or few (level: high) options for finding a male romantic partner; that is, the stimulus indicated that the sex ratio at the university was either male-dominated (e.g. “*almost 65% of on-campus students at the University of Melbourne will be men by 2030*”), or female-dominated (e.g. “*almost 65% of on-campus students at the University of Melbourne will be women by 2030*”), respectively. These primes were improved from Study 1 in that they primed Study 2 participants with information pertaining to their immediate social environment.

##### Attention check

Participants answered five attention check questions (e.g. “*how easy would it be for a female student at The University of Melbourne to find love?*”) using free-text responses. Participants who responded incorrectly to more than one question were excluded from the study. This attention check was intended to operate not only as a means to exclude non-attentive participants, but also as a means through which attentive participants could better engage with and comprehend the magazine article stimulus. By using five free-text responses in Study 2 in place of one multiple choice response (as per Study 1), we aimed to elicit greater engagement and comprehension.

##### Manipulation check

Participants completed a single-item manipulation check: “*After reading the above article, to what extent do you think that you would need to try harder if you wanted to outcompete other women at The University of Melbourne?*” Responses were measured on a five-point Likert scale (1 = not at all, 5 = to a great extent; *M* = 2.54, *SD* = 1.20). This question was adapted from Griskevicius et al. (2012). It is better suited to measuring state intrasexual competitiveness (relative to the Intrasexual Competition Scale used in Study 1) because participants are not required to reflect on their competitiveness outside of the experimental scenario or to examine their absolute beliefs.

##### Other measures

Sex-is-power-belief, relative attractiveness, derogation, and social exclusion were measured in the same way as in Study 1.

##### Descriptive statistics for measures

See Table 2 for measure descriptive statistics.

**Table 2.** Descriptive statistics for measures included in study 2

Scale	Subscale	Mean	SD	*α*
**Sex-is-power belief**		4.16	0.85	0.85
**Relative attractiveness of the recipient**		3.53	0.91	NA
**Derogation**	Warmth and morality	3.08	0.53	0.83
	Warmth but not morality	3.43	0.51	0.76
	Morality but not warmth	3.21	0.53	0.80
	Competence	3.06	0.53	0.82
**Social exclusion**		4.12	0.86	0.85

#### Procedure

**Figure 2.**
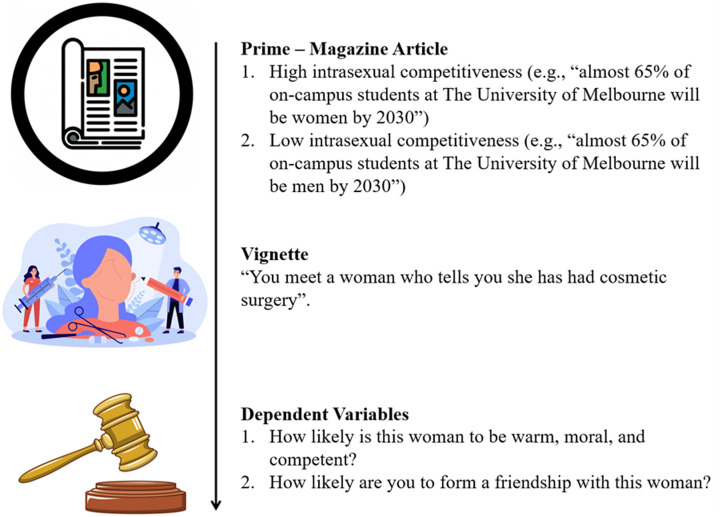
(Abridged) procedure for Study 2.

After obtaining consent, participants were told that they would be presented with a magazine article written by researchers at The University of Melbourne in an attempt to educate the broader public about sex ratios on university campuses. Participants were told that after reading the article they would complete an article comprehension task to help researchers understand whether the article was ready for publication. At this point, participants were randomly presented with either the low or high intrasexual competitiveness priming article. Next, participants were presented with the attention check (framed to them as a comprehension task) and the manipulation check. Participants then completed the sex-is power belief scale. Afterwards, they were presented with the vignette and asked to indicate the relative attractiveness of the recipient. Participants then completed the derogation and social exclusion measures. See Figure 2 for a summary of the major components (i.e., prime and dependent variables) of our procedure.

#### Statistical analyses

**Figure 3.**
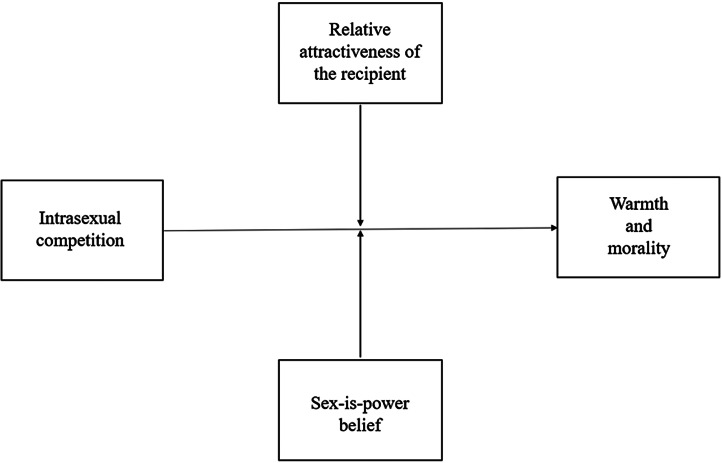
Model 1: moderation analysis with intrasexual competition as the independent variable (IV), warmth and morality as the dependent variable (DV), and relative attractiveness of the recipient and sex-is-power belief as moderators.

**Figure 4.**
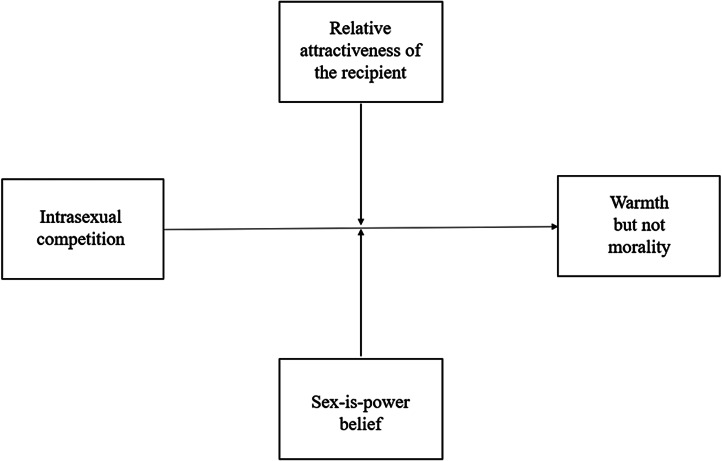
Model 2: moderation analysis with intrasexual competition as the IV, warmth but not morality as the DV, and relative attractiveness of the recipient and sex-is-power belief as moderators.

**Figure 5.**
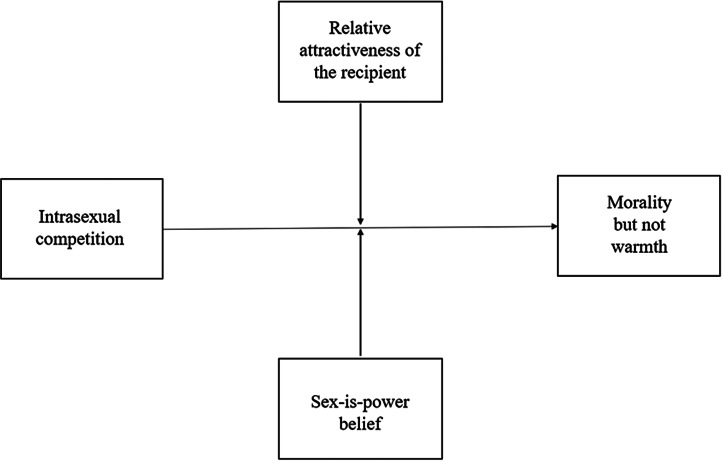
Model 3: moderation analysis with intrasexual competition as the IV, morality but not warmth as the DV, and relative attractiveness of the recipient and sex-is-power belief as moderators.

**Figure 6.**
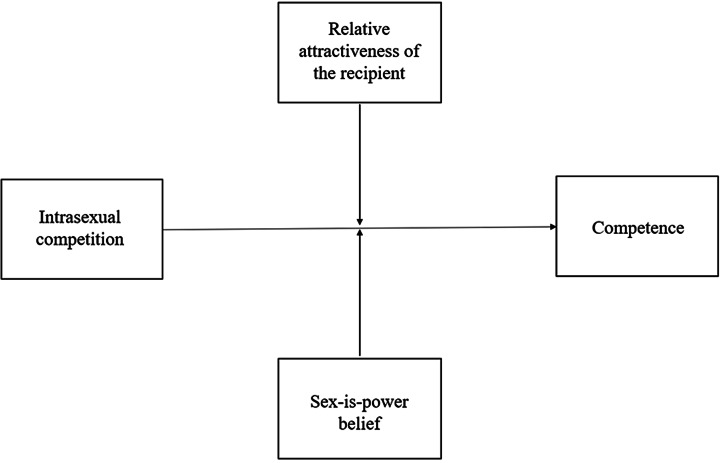
Model 4: moderation analysis with intrasexual competition as the IV, competence as the DV, and relative attractiveness of the recipient and sex-is-power belief as moderators.

**Figure 7.**
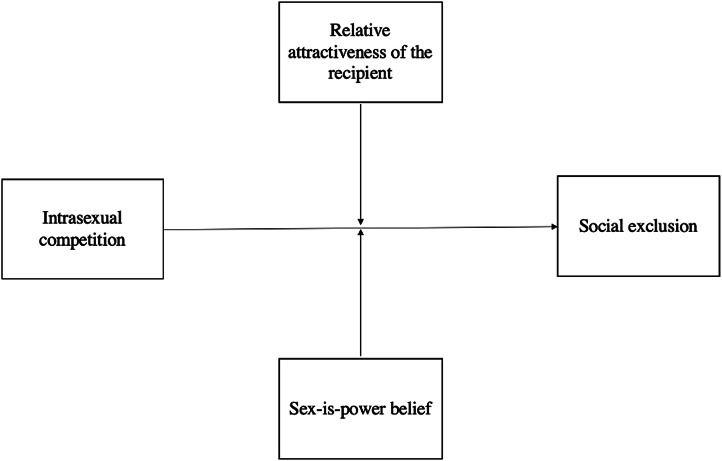
Model 5: moderation analysis with intrasexual competition as the IV, social exclusion as the DV, and relative attractiveness of the recipient and sex-is-power belief as moderators.

Once again, we conducted a manipulation check using the same analysis outlined in Study 1. We then used Hayes’ PROCESS macro in SPSS to run moderation analyses (using the ‘Model 2’ function). We examined (i) the effect of the priming condition on mean differences for degradation and social exclusion and (ii) whether the size of these effects differed as a function of the relative attractiveness of the recipient and/or participant sex-is-power beliefs (see Figures 3–7). As aforementioned, derogation was operationalised as an umbrella term encompassing (i) warmth and morality, (ii) warmth but not morality, (iii) morality but not warmth, and (iv) competence. Thus, Models 1–4 (Figures 3–6) all represent different operationalisations of derogation as the dependent variable. Conversely, Model 5 (Figure 7) represents social exclusion as the dependent variable.

We applied Bonferroni corrections to all our analyses to account for multiple comparisons; results were only considered significant if *p* < 0.01. We used SPSS to conduct analyses for Study 2. Data and code are available on the Open Science Framework (https://tinyurl.com/2pymy5z6).

#### Power analysis

As per Study 1.

### Results

#### Manipulation check

The manipulation for intrasexual competitiveness was successful: intrasexual competitiveness differed significantly between participants in the low (*M*_low_ = 2.22, SD_low_ = 1.04) and high (M_high_ = 2.87, SD_high_ = 1.26) intrasexual competitiveness prime conditions, *p* < 0.001.

#### Main and exploratory analyses

**Figure 8.**
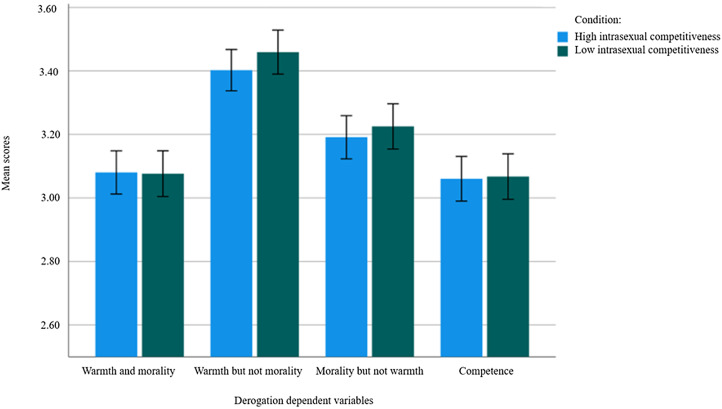
Mean differences on derogation dependent variables as a function of priming condition (including 95% confidence interval [CI] error bars).

**Figure 9.**
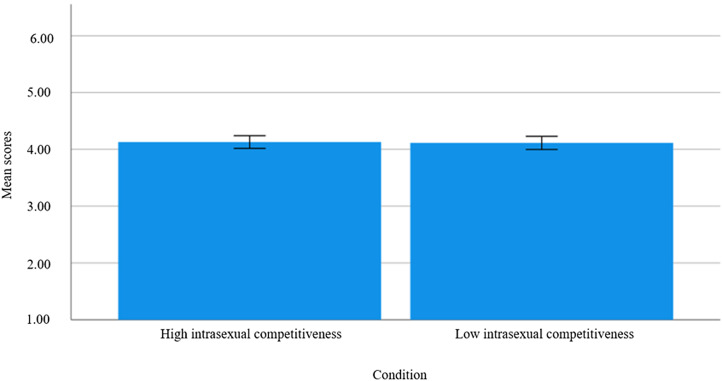
Mean differences on social exclusion as a function of priming condition (including 95% CI error bars).

We assessed Models 1–5 as outlined in Figures 3–7. In assessing Models 1–4, we determined that there was no direct effect of intrasexual competitiveness on derogation (operationalised via warmth and morality, *b* = 0.18, *p* = 0.551; warmth but not morality, *b* = 0.13, *p* = 0.644; morality but not warmth, *b* = 0.26, *p* = 0.393; competence, *b* = −0.11, *p* = 0.720) when accounting for both our proposed moderators (see Figure 8). In assessing Model 5, we determined there was also no direct effect of intrasexual competitiveness on social exclusion (*b* = 0.76, *p* = 0.121) when accounting for both our proposed moderators (see Figure 9).

In Models 1–4, relative attractiveness did not moderate the relationship between intrasexual competitiveness and any of the four operationalisations for derogation (warmth and morality, *b* = −0.04, *p* = 0.427; warmth but not morality, *b* = −0.01, *p* = 0.841; morality but not warmth, *b* = −0.02, *p* = 0.708; competence, *b* = −0.05, *p* = 0.344). Likewise, sex-is-power belief did not moderate the relationship between intrasexual competitiveness and any of the four operationalisations for derogation (warmth and morality, *b* = −0.01, *p* = 0.885; warmth but not morality, *b* = −0.04 *p* = 0.447; morality but not warmth, *b* = −0.06, *p* = 0.329; competence, *b* = 0.07, *p* = 0.267).

For Model 5, neither relative attractiveness (*b* = −0.11, *p* = 0.224) nor sex-is-power belief (*b* = −0.09, *p* = 0.334) moderated the relationship between intrasexual competitiveness and social exclusion.

Across all models, however, there was a main effect for relative attractiveness – relative attractiveness predicted scores on all the derogation outcome variables (warmth and morality, *b* = 0.12, *p* = 0.004; warmth but not morality, *b* = 0.16, *p* < 0.001; morality but not warmth, *b* = 0.14, *p* < 0.001; competence, *b* = 0.15, *p* < 0.001) and on social exclusion (*b* = 0.26, *p* < 0.001). In other words, the cosmetic surgery recipient faced less derogation and social exclusion (i.e., was rated higher on warmth and morality, wamrth but not morality, morality but not warmth, competence, and social inclusion) the more attractive participants presumed her to be compared with themselves.

### Discussion

This study was the first to examine whether negative attitudes towards cosmetic surgery recipients are driven (at least in part) by intrasexual competitiveness. In the context of our manipulations and methods, intrasexual competitiveness did not significantly drive participants to derogate or socially exclude women who undergo cosmetic surgery. Thus, our primary hypothesis was not supported. Similarly, our exploratory hypotheses were not supported by results. The relationships between prime condition and derogation, and prime condition and social exclusion, were not moderated by the relative attractiveness of the recipient or by participants' sex-is-power beliefs.

The fact that our primary hypotheses was not supported might suggest that state-level manipulations (i.e. manipulations meant to induce certain feelings *in the given moment*) are simply not powerful enough to ‘override’ established attitudes towards cosmetic surgery. In other words, if an individual fundamentally supports cosmetic surgery, inducing state-level intrasexual competitiveness may not subdue these beliefs; conversely, if an individual already condemns cosmetic surgery, priming them for low intrasexual competitiveness may not placate this belief. To test this theory, we recommend future literature employ within-subject designs (as opposed to our between-subjects design) to determine whether state-level manipulations have any impact on individuals’ beliefs towards cosmetic surgery. We also recommend that future research examines whether trait intrasexual competitiveness (i.e. participants’ baseline levels of intrasexual competitiveness) predicts attitudes towards cosmetic surgery recipients, for example, whether participants’ scores (sans manipulation) on the Intrasexual Competition Scale (Buunk & Fisher, 2009) predict derogation and social exclusion.

Conversely, our non-significant findings may simply suggest that intrasexual competitiveness does not influence cosmetic surgery attitudes. Being primed for intrasexual competition may only alter one's attitudes towards cosmetic surgery if surgery itself is construed as an act that enhances one's mate value and makes one more competitive (i.e. more attractive to the opposite sex). Our findings suggest that cosmetic surgery may simply not be associated with mate value (i.e. participants may not see undergoing cosmetic surgery as necessarily indicative of becoming more attractive). This could reflect participants' understanding that cosmetic surgery is stigmatised and thus perhaps not desirable in a potential mate (even if it does promote attractiveness).

Alternatively, the results may suggest that while cosmetic surgery is indeed associated with mate value, mate value itself may not inherently prompt derogation and social exclusion. While participants prompted for high intrasexual competitiveness may have viewed the cosmetic surgery recipient as inherently more threatening than those prompted for low competitiveness, behavioural responses to this threat may have differed between participants. For example, while some women might socially exclude perceived sexual threats from their social networks, others might act inclusively in an attempt to benefit from social proximity to these women. Thus, perhaps we saw no significant main effect for priming condition on derogation and social exclusion because not all women derogate or socially exclude their ‘competition’; some women may admire and befriend those that they consider intimidating. Finally, the degree to which intrasexual competitiveness informs the derogation and social exclusion of cosmetic surgery recipients may depend on the type of surgery someone is getting. Enhancing secondary sexual characteristics might be considered differently to enhancing non-sexual characteristics, for example, as these characteristics may be associated with greater perceptions of mate value. Our failure to account for these differences may explain our lack of significant findings.

We propose that, ultimately, alternative explanations for cosmetic surgery recipient derogation and social exclusion need be examined. Previous research suggests that negative attitudes towards cosmetic surgery may be driven by perceptions that cosmetic surgery is unnatural (Bonell et al., 2020; Bonell et al., 2021a, 2021b). In line with theoretical literature that suggests naturalness is often equated with morality (Takala, 2004), Bonell et al. (2020) found that valuing ‘naturalness’ predicts perceptions that cosmetic surgery is immoral. In the present study, recipient derogation was measured (in part) as pertaining to morality. Therefore, perceptions of naturalness may drive recipient derogation. Future research ought to examine this possibility.

#### Relative attractiveness: a novel construct and novel findings

While our hypotheses were generally not supported, we did find that relative attractiveness negatively predicted derogation and social exclusion. These results corroborate the existing attractiveness literature that suggests attractive individuals are generally treated better and are more likely to be ascribed prosocial characteristics (i.e. the ‘halo effect’ or ‘pretty privilege'; Dion et al., 1972; Eagly et al., 1991; Wheeler & Kim, 1997). However, to the authors’ knowledge, our conceptualisation of attractiveness (i.e. as relative to participants’ own attractiveness) was novel. In other words, past studies have established that, on average, ‘more attractive’ people are treated more kindly by others than ‘less attractive’ people (Bonell et al., 2021b; Dion et al., 1972; Eagly et al., 1991; Wheeler & Kim, 1997). However, our study is the first to demonstrate that simply being relatively more attractive than someone has implications for the way they are likely to treat you. As such, we propose that future research examines the role relative attractiveness plays in attractiveness biases more broadly.

## Conclusions

We examined whether state-level intrasexual competitiveness would drive negative attitudes towards cosmetic surgery recipients. Overall, our hypotheses were not supported. We concluded that intrasexual competitiveness may only influence cosmetic surgery attitudes at a trait level and/or that other mechanisms may simply better account for negative cosmetic surgery attitudes. Furthermore, while tangential to our aims, we noted that findings from the present study contribute substantially to existing attractiveness literature. We recommend that future literature continues to examine the mechanisms through which cosmetic surgery recipients come to face derogation and social exclusion.

## Data Availability

Data and code are available on the Open Science Framework (https://tinyurl.com/2pymy5z6).

## Research transparency and reproducibility

Data and code are available on the Open Science Framework (https://tinyurl.com/2pymy5z6).
